# Osteogenic and anti-inflammatory effect of the multifunctional bionic hydrogel scaffold loaded with aspirin and nano-hydroxyapatite

**DOI:** 10.3389/fbioe.2023.1105248

**Published:** 2023-01-24

**Authors:** Shaoping Li, Yundeng Xiaowen, Yuqing Yang, Libo Liu, Yifan Sun, Ying Liu, Lulu Yin, Zhiyu Chen

**Affiliations:** ^1^ Key Laboratory of Stomatology in Hebei Province, Hospital of Stomatology Hebei Medical University, Shijiazhuang, China; ^2^ College of Dentistry, Hebei Medical University, Shijiazhuang, China

**Keywords:** aspirin, multifunctional hydrogel scaffold, sustained release, tissue engineering, nano-hydroxyapatite

## Abstract

Although tissue engineering offered new approaches to repair bone defects, it remains a great challenge to create a bone-friendly microenvironment and rebuild bone tissue rapidly by a scaffold with a bionic structure. In this study, a multifunctional structurally optimized hydrogel scaffold was designed by integrating polyvinyl alcohol (PVA), gelatin (Gel), and sodium alginate (SA) with aspirin (ASA) and nano-hydroxyapatite (nHAP). The fabrication procedure is through a dual-crosslinking process. The chemical constitution, crystal structure, microstructure, porosity, mechanical strength, swelling and degradation property, and drug-release behavior of the hydrogel scaffold were analyzed. Multi-hydrogen bonds, electrostatic interactions, and strong “egg-shell” structure contributed to the multi-network microstructure, bone tissue-matched properties, and desirable drug-release function of the hydrogel scaffold. The excellent performance in improving cell viability, promoting cell osteogenic differentiation, and regulating the inflammatory microenvironment of the prepared hydrogel scaffold was verified using mouse pre-osteoblasts (MC3T3-E1) cells. And the synergistic osteogenic and anti-inflammatory functions of aspirin and nano-hydroxyapatite were also verified. This study provided valuable insights into the design, fabrication, and biological potential of multifunctional bone tissue engineering materials with the premise of constructing a bone-friendly microenvironment.

## 1 Introduction

The implant denture is a superior method to restore lost teeth, oral function, and aesthetics. In clinic, severe and large-sized jaw and alveolar bone defects are often secondary to trauma, periodontal disease, tumors, etc., (Esposito et al., 2006). Bone augmentation surgery is indispensable to ensure adequate and stable implant osseointegration in this situation (Schmitz and Hollinger, 1986; Hegde et al., 2016). In the bone tissue engineering field, increasing experimental evidence has confirmed that the application of bioactive synthetic materials is superior to several conventional methods (e.g autologous bone, allogeneic bone, and artificial bone meal filling), for eliminating the restraint of limited sources of donors, secondary trauma, pathogen transmission, and immune rejection (Li et al., 2018). Recent studies have reported that inflammatory and reconstructive microenvironment resulting from microtrauma and the body’s physiological adaptation instincts can lead to the decline of osteoblast function (Fasolino et al., 2019). Several studies have suggested that bone-friendly bioactive materials may accelerate bone regeneration, but it remains a challenge (Zhang et al., 2022). Hence, it is of great clinical and scientific value to develop a high-quality bone tissue engineering material that can outperform currently available protocols.

Hydrogels have been widely applied to facilitate functional and constructive tissue repair in many clinical applications. Invaluable qualities such as interconnected porous morphology and architecture similar to extracellular matrix (ECM), irreplaceable water retention capacity, drugs and growth factors delivery effect, and biodegradability enable hydrogel to adapt to specific physical and soft tissue regeneration environment (Han et al., 2021; Sreekumaran et al., 2021; Sun et al., 2022). Furthermore, some attention has been fortunately drawn to the roles of the non-steroidal anti-inflammatory drug (NSAIDs) of aspirin, since it is used to alleviate the inflammation and activate osteoblasts in the surgical site within the concentration range of 50–300 μg/mL (Fattahi et al., 2022). Studies have confirmed that an appropriate dose of aspirin can improve trabecular bone structure, bone mineral density, and bone mechanical strength (Tao et al., 2020). Low-dose aspirin can promote bone marrow mesenchymal stem cells to change into osteoblasts and accelerate bone regeneration (Xie et al., 2019). Previous literature has reported that local injection of aspirin also can reduce Interferon γ (IFN-γ) and tumor necrosis cytokines (TNF-α) in the bone defect area (Liu et al., 2018). However, to maintain effective concentration and efficacy in the defect area, systemic high doses drug application is inevitably required because of the short half-life of aspirin (Bliden et al., 2016; Tao et al., 2019), which may lead to adverse effects such as liver and kidney damage and gastrointestinal bleeding (CAVAGNI et al., 2016). It is a viable option that the application of hydrogel scaffolds as a platform to release aspirin. So far, many efforts have been taken to explore an ideal hydrogel for bone tissue engineering. Yet, the current structure and properties are still unsatisfactory, whether prepared by a single component or single cross-linking method.

Gelatin (Gel) is temperature-sensitive, and is a partial hydrolysis product of native collagen. Sodium alginate (SA) is a natural polysaccharide derived from brown seaweed that has been extensively applied in tissue engineering to deliver drugs and growth factors (Fang et al., 2022; Tan et al., 2022). Moreover, SA can be chemically cross-linked by Ca^2+^ (Wang et al., 2019). There is a strong electrostatic interaction between Gel and SA when they are mixed and cross-linked (Xu et al., 2019). Additionally, polyvinyl alcohol (PVA) stands out from synthetic organic compounds due to its excellent water solubility and stable chemical properties (Ahmed et al., 2021). Since there are a large number of free hydroxyl groups, the PVA is tightly connected through hydrogen bonds when treated by cyclic freeze-thaw (Ahmed et al., 2021).

To warrant optimal behavior, especially the mechanical strength and bone repair capacity, the desired quality of the bone tissue engineering scaffold involves adding inorganic bioceramic components (Patel et al., 2019). Nano-hydroxyapatite (nHAP) is a reasonable choice as it has similar chemical compositions to natural bone. And it is regarded as a promising material due to its outstanding osteoconductivity, osteoinductivity, drug-loading property, and suitable cell adhesion property (Raina et al., 2020; Jiang et al., 2021). Furthermore, the incorporation of hydroxyapatite nanoparticles into a hydrogel may also provide a possibility to overcome its disadvantages of high brittleness, morphological instability, and slow degradation (Pina et al., 2015; Chocholata et al., 2020), simultaneously, improving the mechanical strength and characterizations of the hydrogel (Wang et al., 2020; Karimipour-Fard et al., 2021). Yet, the impact of aspirin and nano-hydroxyapatite on hydrogel scaffolds, and whether they have a synergistic function, is still unclear.

**SCHEME 1:**
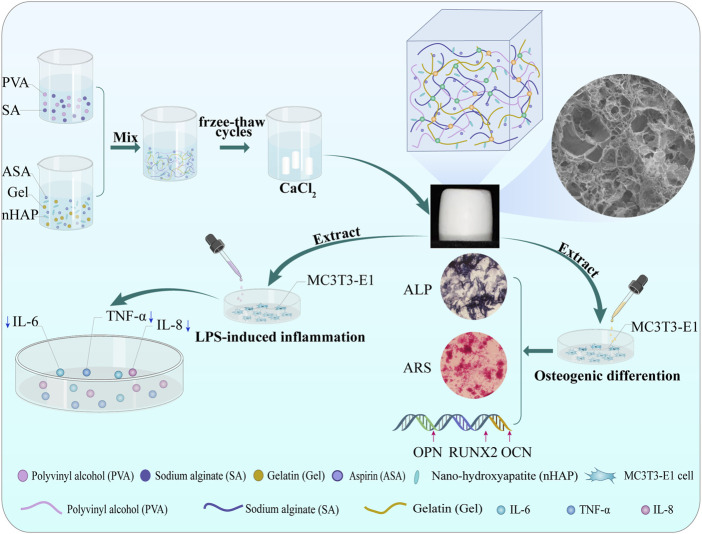
Schematic illustration of the procedures of the dual-crosslinking hydrogel scaffolds. Effects on MC3T3-E1 cells bioactivity, osteogenic differentiation, and anti-inflammatory capacity.

This study proposed a novel strategy for preparing a multifunctional and highly biomimetic hydrogel scaffold. A hydrogel scaffold by a combination of PVA, SA, and Gel with the addition of aspirin and nano-hydroxyapatite was successfully constructed by cyclic freeze-thawing and soaking in calcium chloride (CaCl_2_) solutions. This method is practical, economical, and efficient. Subsequently, the hydrogel scaffold was characterized by Fourier Transform Infrared Spectroscopy (FTIR), X-ray Diffraction (XRD), and Scanning Electron Microscope (SEM) to confirm the chemical constitution, crystal structure, and microstructure. The porosity, mechanical property, swelling, degradation and drug release behavior of the hydrogel scaffold were investigated *in vitro*. The biocompatibility such as cytotoxicity, cell proliferation, cell adhesion behavior, and the osteogenic differentiation ability of alkaline phosphatase (ALP) activity, the expression of calcium nodules and osteogenesis-related genes, as well as the anti-inflammatory effect of the prepared hydrogel scaffold was evaluated using mouse preosteoblasts (MC3T3-E1) cells (Scheme 1).

## 2 Materials and methods

### 2.1 Materials

Polyvinyl alcohol (PVA-124) was purchased from GHTECH (Guangdong, China). Aspirin (ASA), gelatin (Gel, type B), sodium alginate (SA), and anhydrous calcium chloride (CaCl_2_) were obtained from Aladdin (Shanghai, China); nano-hydroxyapatite particle (nHAP, size = 20 nm) was purchased from EMPEROR NANO (Nanjing, China). Phosphate buffer (PBS) and penicillin and streptomycin mixture (100×) were acquired from Solarbio (Beijing, China); fetal bovine serum (FBS) and ɑ-MEM medium were purchased from Biological Industries (Israel). ELISA kits were purchased from Inova (Wuhan, China). Other reagents and solvents were commercially obtained and used as received.

### 2.2 Preparation of the hydrogel scaffold

All solutions were prepared in ultrapure water. Briefly, 1 g of PVA powder was dissolved in 30 mL ultrapure water and constantly agitated at 95°C until completely dissolved to acquire PVA solution. To prepare the PVA-SA solution, when the PVA solution reached a temperature of 37°C, 1 g of SA powder was added and mixed. Meanwhile, 2 g of Gel, 200 μg of aspirin, and 5 g of nano-hydroxyapatite were mixed in 20 mL of ultrapure water to prepare Gel-ASA, Gel-nHAP, and Gel-ASA-nHAP solutions, respectively. Next, various solutions were thoroughly mixed. Air bubbles and the undissolved nano-hydroxyapatite in the hydrogel solutions were removed and shattered with an ultrasonic cleaner for 30 min. Each group of the hydrogel solutions was charged into moulds, frozen at −20°C for 18 h, and then thawed at room temperature for 4 h. The chemical cross-link was carried out after three freeze-thaw cycles, which involved submerging them in 2% CaCl_2_ for 24 h. The hydrogel scaffolds were rinsed with ultrapure water for three times, irradiated, and sterilized at ^60^Co to produce ASA/PVA/Gel/SA, nHAP/PVA/Gel/SA, and ASA-nHAP/PVA/Gel/SA hydrogel scaffolds, named ASA group, nHAP group, and ASA-nHAP group, respectively.

### 2.3 Chemical constitution and crystal structure

#### 2.3.1 FTIR

An IS 50 Fourier Transform Infrared Spectroscopy (FTIR: Thermo, United States) with a resolution of 4 cm^−1^ was used to evaluate the chemical constitution and functional groups in the hydrogel scaffold. At room temperature, the raw materials and freeze-dried hydrogel scaffolds were pulverized and mixed completely with the proper amount of KBr, respectively, before being compacted into tablets. The data of the FTIR spectra were obtained in the range of 4,000–400 cm^−1^ with 32-times scan and then analyzed by Origin 2021 ware.

#### 2.3.2 XRD

The hydrogel scaffolds were compressed as described in 2.3.1 and scanned using a Bruker D8 X-ray Diffractometer (XRD: Advance, Germany). A speed of 2°/min between 10° and 70° was set to acquire the data. The physical phase of the raw materials and hydrogel scaffolds were analyzed by X'Pert High Score software and Origin 2021 ware.

### 2.4 Microstructure

To observe the 3D porous structure of the hydrogel scaffolds, before gold-sputtered, the freeze-dried hydrogel scaffolds were cut in liquid nitrogen. The S-4800 Scanning Electron Microscope (SEM: Hitachi, Japan) was used to observe the microstructure of the hydrogel scaffolds. The imposed accelerating voltage was 15 kV, and the different pore sizes were analyzed.

### 2.5 Characterization of the hydrogel scaffold

#### 2.5.1 Porosity

The porosity was calculated by the liquid displacement method. Briefly, the hydrogel scaffolds (15 mm in diameter, 2 mm in thickness, *n* = 3) were completely immersed in a volume V1 of anhydrous ethanol. After 48 h, the volume V2 was recorded when the hydrogel scaffolds should be completely immersed in anhydrous ethanol, and the liquid surface was free of air bubbles. Subsequently, the hydrogel scaffolds were removed, and the remaining anhydrous ethanol volume V3 was recorded. Porosity was calculated using the following formula: P (%) = (V1–V3)/(V2–V3) × 100%.

#### 2.5.2 Mechanical property

The maximum compression strength and compression modulus of the hydrogel scaffolds (8 mm in diameter, 10 mm in height, *n* = 3) were tested by an RGM-2100 Universal Mechanical Testing Machine (Rigel, Guangzhou, China). The compression test was carried out with a 10 mm diameter flat probe at a speed of 5 mm/min. Compression tests were performed along the long axis of the hydrogel scaffolds at room temperature and 52% relative humidity. When the deformation reached 100%, the compression test was stopped. Then the stress-strain curve and the maximum compression strength were obtained. The compression modulus was calculated as the slope of the linear region of the stress-strain curve.

#### 2.5.3 Swelling property

The swelling performance was examined by calculating the water absorption rate in PBS. Initially, the freeze-dried hydrogel scaffolds (15 mm in diameter, 2 mm in height, *n* = 3) were weighed of W0. Subsequently, the hydrogel scaffolds were immersed in 3 mL of PH = 7.4 PBS at 37°C. The hydrogel scaffolds were removed at different time points, and the surface water was gently wiped. And then they were weighed again of W1 until they reached swelling equilibrium. The swelling property was measured using the formula: swelling rate (%) = W1-W0/W0×100%.

#### 2.5.4 *In vitro* degradation property

To study the *in vitro* degradation property, the freeze-dried hydrogel scaffolds (8 mm in diameter, 10 mm in height, *n* = 3) with the initial weight of W0 were immersed in 4 mL of PBS. Then, the hydrogel scaffolds were placed at 37°C. After 7, 14, 21, and 28 days, they were removed and weighted again of W1 after being freeze-dried. The PBS was changed every 2 days. The degradation rate was evaluated by calculating its weight loss rate with the following formula: degradation rate (%) = (W0-W1)/W0×100%.

#### 2.5.5 *In vitro* aspirin release property

The *in vitro* release of aspirin from the hydrogel scaffolds was estimated. Firstly, the absorbance of aspirin standard working solutions was measured at 274 nm using a TU-1950 Double-beam UV Spectrophotometer (Persee, Beijing, China) for 0, 12.5, 25, 50, 100, 200, and 400 (μg/mL). Next, the standard curve of aspirin was plotted, and the regression equation was calculated (Supplementary Figure S1). Subsequently, the hydrogel scaffolds (*n* = 3) were immersed in 10 mL of PBS at 37°C. Then, 3 mL of liquid was aspirated and centrifuged at corresponding time points respectively. Consequently, 2 mL of supernatant was used to measure the absorbance at 274 nm. Finally, it was replenished with fresh PBS to 10 mL. The cumulative release property of aspirin from the hydrogel scaffold was analyzed by Origin 2021 ware.

### 2.6 Cell growth study

The MC3T3-E1 cells obtained from puonuosai (Wuhan, China) were cultured with a complete medium (CM, containing 10% FBS, and 1% penicillin and streptomycin) in a humidified atmosphere containing at 37°C. All cells were cultured up to the third generation for the subsequent experiments. It was crucial to obtain extraction solutions of the hydrogel scaffolds. Briefly, the ASA, nHAP, and ASA-nHAP group were immersed in CM at a ratio of 10 mg/mL for 24 h. Subsequently, the extractions were filtered by a 0.22 μm filter and stored at 4°C for the next use. For the cytotoxicity and cell proliferation assay, the CM group was used as a control, and the ASA, nHAP, and ASA-nHAP group were regarded as the experiment groups.

#### 2.6.1 Cell viability assay

The cytotoxicity was assessed with a Live/dead Staining Kit (Solarbio, Beijing, China). The MC3T3-E1 cells were seeded in a 96-well plate at a density of 3,000 per well (*n* = 3). After 24 h, the original CM was replaced by 100 μL fresh CM, and the extractions of ASA, nHAP, and ASA-nHAP group for further incubation. The original CM and extractions were replaced with 100 μL of live/dead cell staining reagent after 1, 3, and 5 days and stained for 30 min. The stained cells were observed and imaged by a X171 Inverted Fluorescence Microscope (Olympus, Japan), and then analyzed cell viability.

#### 2.6.2 Cell proliferation assay

A Cell Proliferation Kit (CCK-8, Solarbio, Beijing, China) was applied according to the manufacturer’s instructions. The cells were cultured as described in 2.4.1. After 1, 3, and 5 days, the medium was aspirated. And the cells were mixed with 10 μL of CCK-8 and 100 μL fresh CM. After 1 h of incubation, the optical density (OD) value was detected at a wavelength of 450 nm with a SpectraMax M2 Enzyme Marker (Molecular Devices, United States). The cell proliferation effect was evaluated by comparing the OD value.

#### 2.6.3 Cell adhesion assay

The nucleus and cytoskeleton of MC3T3-E1 cells were observed by the FV1200MPE Laser Confocal Microscopy (CLSM, Olympus, Japan) to evaluate the morphology and adhesion behavior of the MC3T3-E1 cells on the ASA, nHAP, and ASA-nHAP group. The hydrogel scaffolds were incubated with CM for 1 day. Next, the cells were seeded on hydrogel scaffolds at a density of 5 × 10^4^. Two days later, the hydrogel scaffolds were rinsed once with PBS. Then, the cells were fixed with 4% paraformaldehyde for 10 min, and permeabilized with 2% Triton X-100 for 30 min, respectively. The cells were then stained by FITC Phalloidim and 4′, 6-diamantine-2-phenylindole (DAPI), (Solarbio, Beijing, China) at 37°C in dark. Finally, the adhesion behavior of MC3T3-E1 cells was observed by CLSM, and the fluorescence staining area was semi-quantitatively analyzed using Image-Pro Plus 6.0.

### 2.7 Cell osteogenic differentiation study

For the osteogenic differentiation assay, the MC3T3-E1 cells were cultured with osteogenic induction medium (ODM), and the hydrogel scaffold extractions of ASA, nHAP, and ASA-nHAP group, which contains 50 M ascorbic acid, 100 M dexamethasones (Solarbio, Beijing, China) and 10 mM β-sodium glycerophosphate (Sigma-Aldrich, United States). And the solution was changed every 3 days. The ODM group was used as a control, and the ASA, nHAP, and ASA-nHAP group was regarded as the experiment groups.

#### 2.7.1 ALP activity assay

ALP, whose expression was assessed according to the protocol of the 5-Bromo-4-Chloro-3-Indolyl phosphate/Nitro Blue Tetrazolium Kit (BCIP/NBT, Beyotime, Shanghai, China), is an early marker of osteogenic differentiation. The MC3T3-E1 cells were inoculated at a density of 5 × 10^4^ per well in 12-well plates (*n* = 3), and the CM was replaced by ODM of the control group, ASA, nHAP, and ASA-nHAP group when their confluence reached 80%. After 2, 7, and 14 days of culture, the cells were fixed with 4% paraformaldehyde (Solarbio, Beijing, China) for 10 min at room temperature. Then the cells were stained with BCIP/NBT ALP staining solutions in dark for 30 min, and finally terminated the reaction with distilled water. The staining intensity and area were observed and photographed by an Inverted Microscope (Olympus, Japan) to evaluate the ALP activity.

#### 2.7.2 Calcium nodule expression assay

The late osteogenic marker was calcium nodules. It was examined according to the instructions of Alizarin Red Staining solution (Beyotime, Shanghai, China). The cells were cultured as described in 2.5.1 for 7, 14, and 21 days. They were fixed with 95% ethanol for 10 min, and the washed by PBS for three times. The staining procedure was terminated with distilled water after adding appropriate Alizarin Red Staining solution for 30 min. Calcium nodules were observed with a XI71 Inverted Microscope (Olympus, Japan). A semi-quantitative analysis of the expression levels of calcium nodules was also carried out. Briefly, 500 μL of 10% Hexadecane-pyridinium Chloride solution (Solarbio, Beijing, China) was added to the plates containing calcium nodules. The liquid was added to 96 wells at 100 ul per well after 15 min (*n* = 3). The OD value was read at 506 nm, and the semi-quantitative calcium nodules in each group were evaluated.

#### 2.7.3 Osteogenesis-related gene expression assay

The expressions of RUNX2 family transcription factor (RUNX2), osteopontin (OPN), and osteocalcin (OCN) were evaluated by qRT-PCR assay. MC3T3-E1 cells were cultured in a 6-well plate at a density of 1 × 10^5^ per well and cultured as before for 14 days. The total RNA was extracted following the manufacturer’s instructions (ZHONGSHI TONTRU, Tianjin, China), and the concentration was calculated by NanoDrop 2000C (Thermal Fisher Scientific, United States). RNA was reversely transcribed to cDNA by the Reverse Transcription Kit (ZHONGSHI TONTRU, Tianjin, China). Then, an ABI Prism 7,500 Real-Time PCR system (Applied Biosystem, United States) was used after the Mix was added according to the instructions of the Fluorescence Quantitative PCR Kit (ZHONGSHI TONTRU, Tianjin, China). GAPDH was a normalized gene, and the control group was set as 1. The relative expression levels of RUNX2, OCN, and OPN were evaluated (*n* = 3) by the 2^-△△CT^ method. Thermo Fisher synthesized the primers, and the sequence is shown in Table 1.

**TABLE 1:** Primer sequences of target genes for qRT-PCR.

Gene description	Forward primer	Reverse primer
GAPDH	GGT​GAA​GGT​CGG​TGT​GAA​CG	CTC​GCT​CCT​GGA​AGA​TGG​TG
RUNX2	GAC​TGT​GGT​TAC​CGT​CAT​GGC	ACT​TGG​TTT​TTC​ATA​ACA​GCG​GA
OPN	AGC​AAG​AAA​CTC​TTC​CAA​GCA​A	GTG​AGA​TTC​GTC​AGA​TTC​ATC​CG
OCN	CTG​ACC​TCA​CAG​ATC​CCA​AGC	TGG​TCT​GAT​AGC​TCG​TCA​AAG

### 2.8 Anti-inflammatory property study

The anti-inflammatory ability of the hydrogel scaffolds was evaluated by the expression of inflammatory cytokines of MC3T3-E1 cells cultured in a lipopolysaccharide (LPS)-induced (Solarbio, Beijing, China) inflammatory environment. MC3T3-E1 cells were cultured in 6-well plate at a density of 2 × 10^5^, and the medium was replaced with fresh CM, ASA group, nHAP group, and ASA-nHAP group extractions after 24 h. After 30 min, LPS was added to each well at 10 μg/mL. The supernatants of each group were collected after being cultured for 1, 3, and 7 days. And the expression levels of tumour necrosis cytokines (TNF-α), interleukin 6 (IL-6), and interleukin 8 (IL-8) were examined according to the recommended procedure of ELISA Kits (Inova, Wuhan, China). The OD value was measured at 450 nm by the RT-6100 Enzyme Marker (Rayto, Shanghai, China). The concentration of inflammatory cytokines was proportional to the OD value.

### 2.9 Statistical analysis

SPSS, version 22 (IBM), was used for statistical analysis, and the results were compared by the one-way analysis of variance (ANOVA). The data were expressed as mean ± standard deviation. Data were considered to be different when *p < 0.05, and significantly different when **p < 0.01. Origin 2021 was used for plotting.

## 3 Results

### 3.1 Chemical constitution and crystal structure analysis

#### 3.1.1 FTIR analysis

**FIGURE 1:**
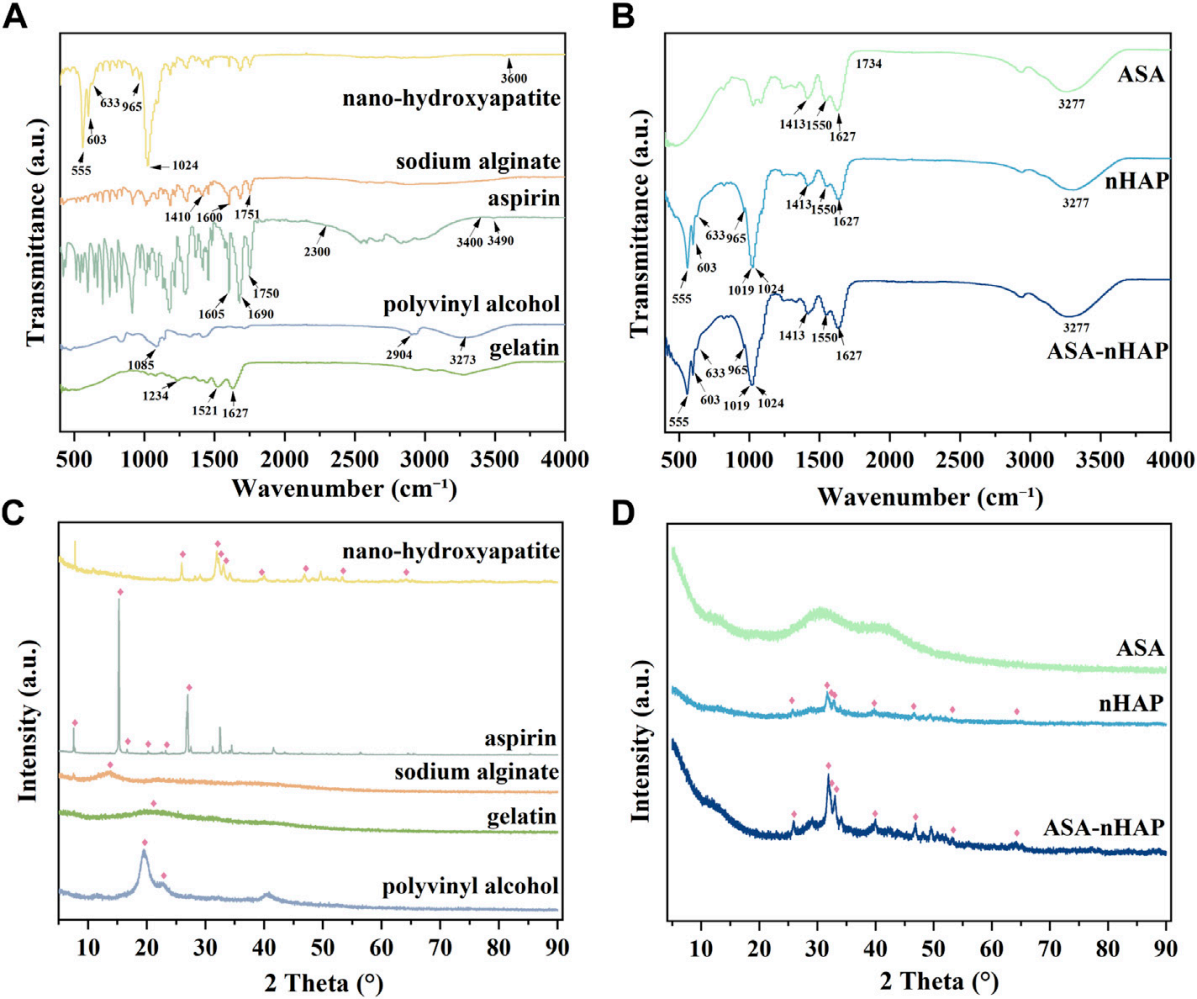
Chemical constitution and crystal structure analysis of the raw powders and the composited hydrogel scaffolds. **(A)** The FTIR spectra of nanohydroxyapatite, aspirin, sodium alginate (SA), gelatin (Gel), and polyvinyl alcohol (PVA). **(B)** The FTIR spectra of ASA/PVA/Gel/SA (ASA group), nHAP/PVA/Gel/SA (nHAP group), and ASA-nHAP/PVA/Gel/SA (ASA-nHAP group) hydrogel scaffold. **(C)** The XRD pattern of nanohydroxyapatite, aspirin, sodium alginate (SA), gelatin (Gel), and polyvinyl alcohol (PVA). **(D)** The XRD pattern of ASA/PVA/Gel/SA (ASA group), nHAP/PVA/Gel/SA (nHAP group), and ASA-nHAP/PVA/Gel/SA (ASA-nHAP group) hydrogel scaffold.

FTIR was used to obtain information on the chemical constitution and related changes of the original powders in hydrogel scaffolds. Figures 1A,B shows the FTIR spectra of the raw material powders and hydrogel scaffolds. As for Gel, its strong absorption peaks amide I band (C=O stretching vibration peak), amide II band (N-H bending vibration peak), and amide III band (C-N stretching vibration peak) located at 1,627 cm^−1^, 1,521 cm^−1^, and 1,234 cm^−1^, respectively. The spectra of PVA showed that the peak at 3,273 cm^−1^, 2,904 cm^−1^, and 1,085 cm^−1^ belonged to the stretching vibration of O-H, C-O, and C-H, respectively. In aspirin, the stretching vibration peaks of -OH presented at 3,490 cm^−1^. The other short peaks in the zone of 2,300–3,400 cm^−1^ were related to -COOH. In addition, the typical peaks of C=O and C=C appeared at 1,690 cm^−1^, 1750 cm^−1^, and 1,605 cm^−1^. The stretching vibration peak of C=O at 1751 cm^−1^. The anti-symmetric and symmetric contraction of -COO- at 1,600 cm^−1^ and 1,410 cm^−1^ were from SA. The prominent peaks of nano-hydroxyapatite posited at 1,024 cm^−1^, 965 cm^−1^, 603 cm^−1^, and 555 cm^−1^ were attributed to the stretching vibration and deformation vibration of PO_4_^3-^. The stretching and bending vibration peaks of -OH were observed at 3,600 cm^−1^ and 633 cm^−1^.

In Figure 1B, the FTIR spectra of the three synthesized hydrogel scaffolds showed the common broad peak at 3,277 cm^−1^, which may correspond to the change and shift of -OH when compared with the raw materials. The amide I bond was significantly broadened at 1,627 cm^−1^, and the amide II band blue-shifted to 1,550 cm^−1^. The weak peaks of aspirin and SA in the fingerprint region also disappeared in the composite hydrogel scaffolds. Another point to notice was that the small peak at 1734 cm^−1^ was related to the shift of C=O, which belonged to SA. These changes in the hydrogel scaffolds proved the formation of hydrogen bonds and strong electrostatic interaction between PVA, SA, Gel, and aspirin (Yang et al., 2020; Zhang et al., 2021). In line with previous studies, the peak at 1,413 cm^−1^ was the “egg-shell” structure formed by the -COO- of SA and Ca^2+^ of the CaCl_2_ (Zhang et al., 2021). The pattern of the nHAP group and ASA-nHAP group showed characteristic peaks at 603 cm^−1^ and 555 cm^−1^. But compared with nano-hydroxyapatite, the peak at 965 cm^−1^ disappeared, and the peak at 1,024 cm^−1^ red-shifted to 1,019 cm^−1^. The reason could be rooted in the formation of hydrogen bonds between PO_4_^3-^ and the raw materials, especially the PVA (Liu et al., 2011; Tohamy et al., 2018). In addition, the difference in intensity of some peaks among the nHAP group and ASA-nHAP group may relate to the presence of aspirin. These results indicated that the physical and chemical cross-linking method was feasible and effective.

#### 3.1.2 XRD analysis

XRD was employed to acquire information on crystalline structure and relevant changes in the hydrogel scaffolds and raw organic powders. The XRD patterns are shown in Figures 1C,D. The characteristic crystalline peaks at 2θ = 19.69° and 2θ = 22° were related to the three-dimensional (3D) porous network in the micro-crystalline regions of the PVA. The Gel and SA were known in the non-crystalline state, their diffuse and broad diffraction peaks at 2θ = 20° and 2θ = 15°, respectively (Yang et al., 2020). Aspirin exhibited some characteristic crystalline peaks at 2θ = 14.37°, 17.3°, 20.0°, 23.5°, and 26.4°. In hydrogel scaffolds, the characteristic diffraction peaks of PVA and aspirin disappeared. And the large and broad diffraction peaks appeared at 2θ = 22.5°–50°, indicating that the organic components interacted with each other during the cross-linking process. Nano-hydroxyapatite displayed some characteristic diffraction peaks at 2θ = 25°, 31.8°, 32.1°, 33°, 39.4°, 46.2°, 53.1°, and 64.1°, which was in agreement with the standard card for nHAP (JCPDS-09–0,432). Peaks of nano-hydroxyapatite in the nHAP group and ASA-nHAP group appeared one by one, indicating that nano-hydroxyapatite was successfully connected to the raw materials. In addition, the intensity of some peaks in the ASA-nHAP group was more obvious than in the nHAP group. A possible explanation was that aspirin successfully attached to nano-hydroxyapatite in the ASA-nHAP group, thus exhibiting stronger diffraction. These results indicated that neither the activity of organic materials nor the crystalline structure of nano-hydroxyapatite was disrupted in the hydrogel scaffolds.

### 3.2 Microstructure analysis

**FIGURE 2:**
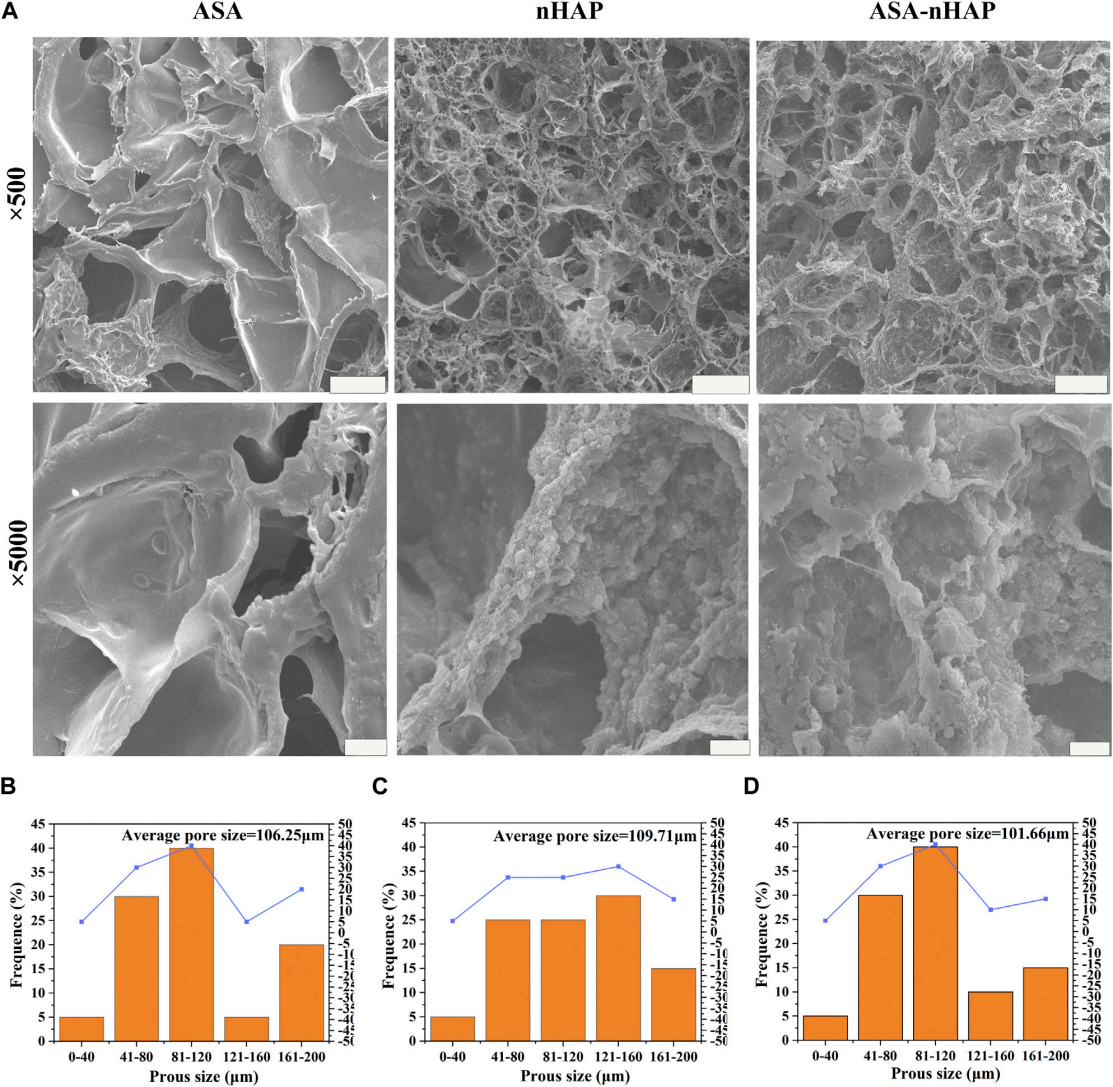
Microstructure of hydrogel scaffolds. **(A)** SEM images of the ASA group, nHAP group, and ASA-nHAP group. The scale bar for low-magnification images is 100 μm; the scale bar for high-magnification images is 10 μm. **(B–D)** Pore size distribution pattern of the ASA group, nHAP group, and ASA-nHAP group.

The microstructure of the hydrogel scaffolds was examined by SEM. As shown in Figure 2A, the images showed a uniform and interconnected distribution of pores in the ASA group. Additionally, smooth pore walls also could be observed. As for the nHAP group and ASA-nHAP group, a pronounced hierarchical multi-network structure appeared when the hydroxyapatite nanoparticles were uniformly and closely attached to the surface of the pore. Compared with the ASA group, the sidewall’s thickness and roughness were increased. Additionally, Figure 2B illustrates the regularities of distribution of the pore sizes of the ASA, nHAP, and ASA-nHAP group. The average pore size was about 106.25 μm, 109.71 μm, and 101.66 μm, respectively. The results showed that aspirin had no significant impact, while nano-hydroxyapatite provided a more reliable microstructure and the application potential of the hydrogel scaffold in bone tissue engineering. Furthermore, it verified that the dual-crosslinking method made a tight association between organic materials and nano-hydroxyapatite.

### 3.3 Characterization analysis

#### 3.3.1 Porosity analysis

**FIGURE 3:**
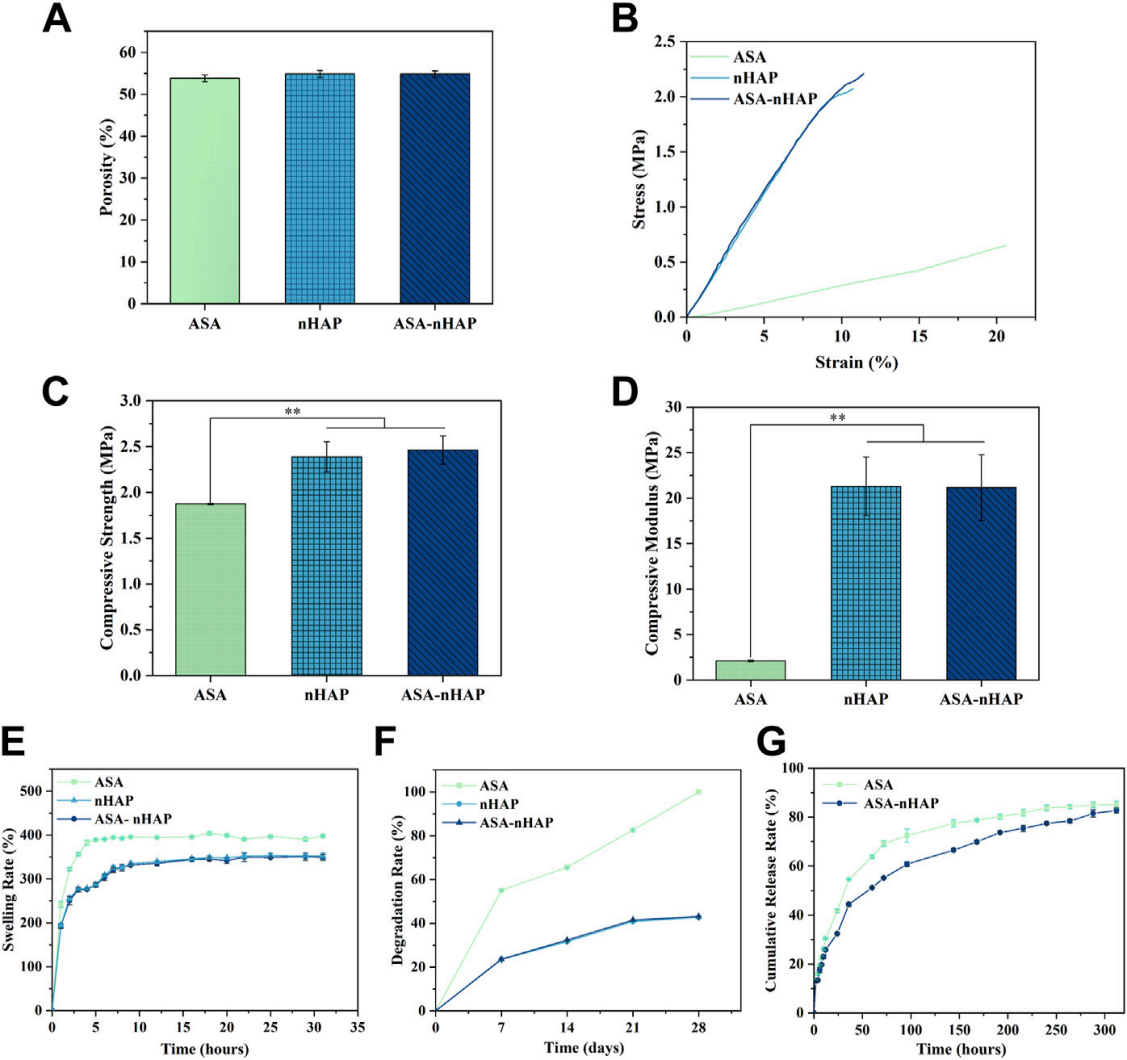
Characterization of hydrogel scaffolds. **(A)** The porosity of hydrogel scaffolds. **(B)** The stress-strain curve of hydrogel scaffold. **(C)** The compressive strength of hydrogel scaffold. **(D)** The compressive modulus of hydrogel scaffold. **(E)** The swelling performance of hydrogel scaffolds. **(F)** The degradation performance of hydrogel scaffolds. **(G)** The drug release performance of hydrogel scaffolds. (**p < 0.01).

Figure 3A shows the porosity of different hydrogel scaffolds. The porosity was similar, and about 53.8 ± 0.82%, 54.87 ± 0.76%, and 54.83 ± 0.84% for the ASA, nHAP, and ASA-nHAP group, respectively (*p* > 0.05). This provided evidence neither aspirin nor nanohydroxyapatite adversely affected the porosity of the hydrogel scaffold. And it was also confirmed that nano-hydroxyapatite adhered tightly to the porous surface.

#### 3.3.2 Mechanical strength analysis

The hydrogel scaffold’s compression strength and compression modulus were evaluated in depth. As shown in Figure 3B, the stress-strain curve of the ASA group had the lowest slope. As for the nHAP and ASA-nHAP group, they were higher and extremely similar. By comparison, with the same test condition, the ASA group exhibited worse mechanical strength. As shown in Figure 3C, the compressive strength of the ASA group, nHAP group, and ASA-nHAP group was about 1.88 ± 0.01 MPa, 2.39 ± 0.16 MPa, and 2.46 ± 0.15 MPa, respectively. Additionally, Figure 3D shows the results of the compression modulus by further analysis of the stress-strain curve. Compared with 2.11 ± 0.08 MPa of the ASA group (*p <* 0.01), the compression modulus of the nHAP and ASA-nHAP group was positively enhanced to 21.16 ± 3.23 MPa and 21.28 ± 3.62 MPa (*p* > 0.05), respectively. These findings hinted that they could withstand compression without breaking. Moreover, the addition of nano-hydroxyapatite could significantly improve the mechanical properties of hydrogels composed by pure organic materials.

#### 3.3.3 Swelling property analysis

As shown in Figure 3E, the ASA group, nHAP group, and ASA-nHAP group had similar swelling profiles and suitable swelling rates in PBS. Conspicuously, the ASA group reached swelling equilibrium as early as 6 h, with a swelling rate of 397.71 ± 1.28%. The ASA group had the highest swelling rate compared to the nHAP and ASA-nHAP group (*p* < 0.01). Also, the swelling rate of the nHAP and ASA-nHAP group was not significantly different, which decreased to 349.18 ± 6.34% and 352.30 ± 6.92% (*p* > 0.05), respectively. Meanwhile, the time to swelling equilibrium was significantly prolonged to 16 h, which suggested they were equipped with more appropriate swelling behavior. The results showed that nano-hydroxyapatite with smaller hydrophilic could reduce the swelling behavior, resulting in a better release of aspirin.

#### 3.3.4 *In vitro* degradation property analysis

The *in vitro* degradation capacity of the hydrogel scaffold was evaluated according to the weight reduction rate when immersed in PBS for a certain period. As shown in Figure 3F, the ASA group degraded more rapidly than the nHAP group and ASA-nHAP group, with a degradation rate of 100% after 28 days (*p <* 0.01). The final degradation rate of the nHAP group and the ASA-nHAP group was significantly lower than the ASA group, and was about 42.72 ± 0.01% and 43.11 ± 0.02%, respectively (*p* > 0.05). The results indicated that the nHAP group and ASA-nHAP group had a more trustworthy stability. Another point according to Figure 3F was that the degradation speed was higher at the first 7 days for the three groups, but then the speed became slower until 28 days, especially the nHAP group and ASA-nHAP group.

#### 3.3.5 *In vitro* drug release property analysis

The drug release property of the hydrogel scaffolds was investigated *in vitro* at 37°C. The drug release rate and profile of the ASA group and ASA-nHAP group are presented in Figure 3G. The ASA group and ASA-nHAP group had excellent drug loading efficiency for their cumulative release rate of 85.13% and 82.75%, respectively. As we can see, a burst release of aspirin happened in the first 48 h in all groups, and the ASA group had a more obvious burst release rate. The burst release presumably resulted from the exchange of water molecules and the breakage of organic polymerization chains. After 48 h, the release speed was gradually reduced and sustained until 312 h, suggesting that the fabricated hydrogel scaffold kept a long time of releasing aspirin. The aspirin release profile of the ASA-nHAP group was flatter after 48 h, which indicated that the ASA-nHAP group had a better-sustained release capacity. This also confirmed the drug-loaded capacity of nano-hydroxyapatite.

### 3.4 Cell growth properties

#### 3.4.1 Cell viability analysis

**FIGURE 4:**
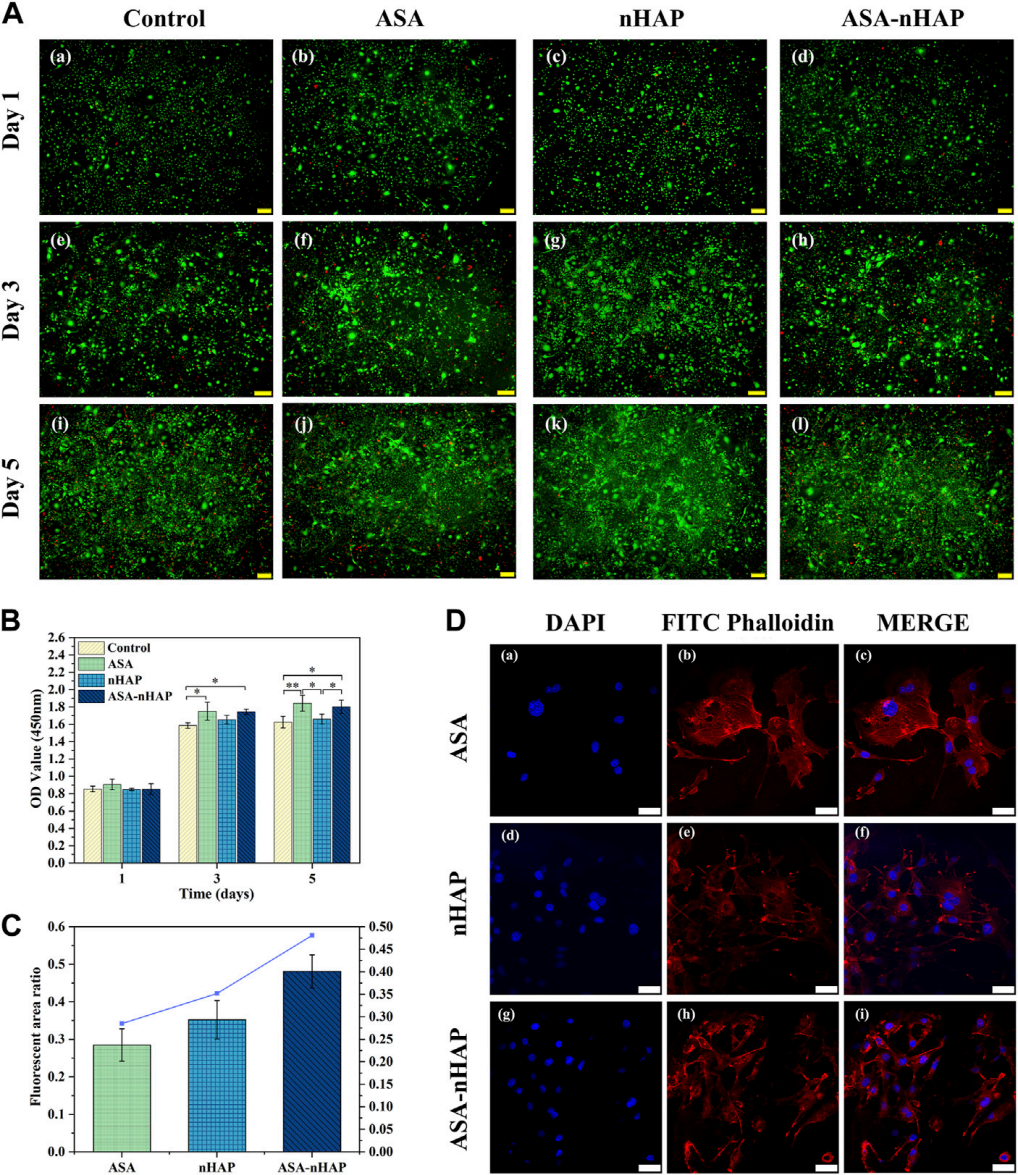
Growth ability of MC3T3-E1 cells cultured *in vitro*. **(A)** Live/dead staining images of MC3T3-E1 cells cultured with the hydrogel extractions for 1, 3, and 5 d. **(B)** The OD value of the control group, ASA group, nHAP group, and ASA-nHAP group from the CCK-8 assay (*p < 0.05, **p < 0.01). **(C, D)** Semi-quantitative analysis and laser confocal images of MC3T3-E1 cells on the ASA group, nHAP group, and ASA-nHAP group. Blue represents the nucleus stained by DAPI and red represents the cytoskeleton stained by FITC phalloidin at a scale bar of 40 μm.

The ability to maintain cell viability is a primary prerequisite for further functional studies and clinical applications of the hydrogel scaffold. Figure 4A shows the viability of MC3T3-E1 cells with live/dead staining after 1, 3, and 5 days. Green fluorescence represented viable cells, and red fluorescence represented dead cells. The cells cultured with different experimental groups exhibited higher viability and growth trend than the control group over time. Furthermore, the images showed a uniform distribution of cells with a polygonal morphology. This proved that the hydrogel scaffolds was no cytotoxicity.

#### 3.4.2 Cell proliferation analysis

The results of the CCK-8 experiment are shown in Figure 4B. The OD value was proportional to cell proliferation. Although there was no significant difference in OD value among all groups on the first day, the OD value increased over time. And the higher OD value in the experimental groups emerged at the 3 days and 5 days. It also could be found that among the three experimental groups, the ASA group had a more significant cell proliferation effect, and the nHAP group had a slightly lower cell proliferation effect. The results indicated that the hydrogel scaffold could promote cell proliferation over time, which was consistent with the results of cytotoxicity experiments.

#### 3.4.3 Cell adhesion analysis

The semi-quantitative and staining results are shown in Figures 4C,D. The actin filament was stained red by FITC Phalloidin, and the nucleus was stained blue by DAPI in CLSM. Interestingly, in different groups, MC3T3-E1 cells strongly attached to the surface, regularly distributed, and formed cell bridges, which suggested that the biocompatibility and the 3D porous structure of the hydrogel scaffold facilitated cell interaction and cell adhesion. A slightly larger number of MC3T3-E1 cells were observed in the nHAP and ASA-nHAP group than in the ASA group. The connection of cell pseudopods was also more intimate, especially in the ASA-nHAP group. These results indicated that the prepared ASA-nHAP group may provide a more bone-friendly microenvironment for the growth of MC3T3-E1 cells.

### 3.5 Cell osteogenic differentiation property

#### 3.5.1 ALP activity analysis

**FIGURE 5:**
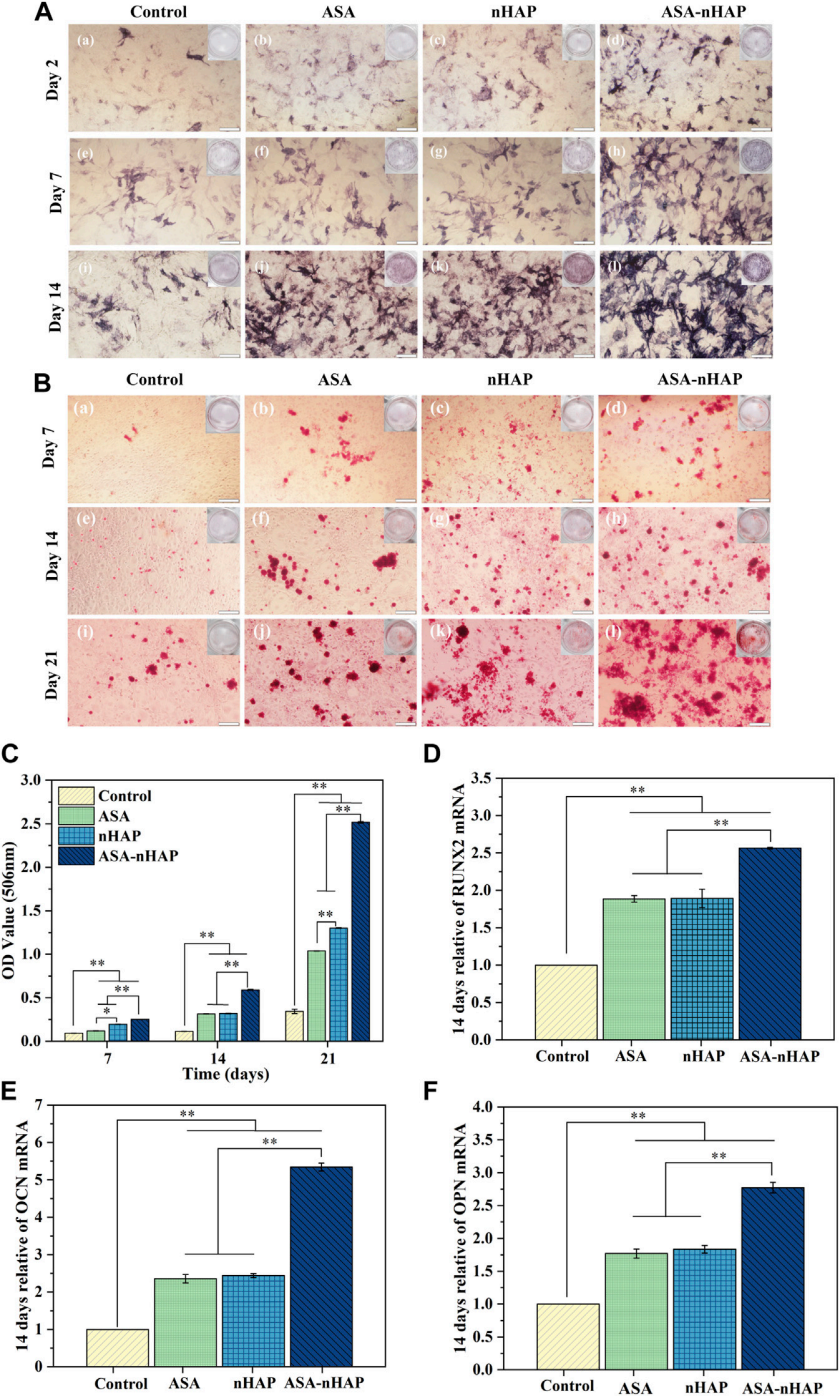
Osteogenic differentiation ability of MC3T3-E1 cells cultured *in vitro*. **(A)** Digital and microscopic images of MC3T3-E1 cells in the control group, ASA group, nHAP group, and ASA-nHAP group after 2, 7, and 14 days to assess ALP activity, the scale bar is 200 μm. **(B)** Digital and microscopic images of MC3T3-E1 cells in the control group, ASA group, nHAP group, and ASA-nHAP group after 7, 14, and 21 days to assess the expression of calcium nodules, scale bar is 200 μm. **(C)** Semi-quantitative analysis of the expression of calcium nodules in the control group, ASA group, nHAP group, and ASA-nHAP group after 7, 14, and 21 days (**p < 0.01). **(D–F)** Expression of osteogenic genes of RUNX2, OCN, and OPN after 14 days (**p < 0.01).

Figure 5A shows the ALP activity of each group. The expression of ALP occurs in the early phase of bone regeneration. ALP activity was evaluated according to the color intensity. The results showed that the ALP activity increased over time in all groups. In contrast to the control group, a significantly visible purple color emerged in the experimental groups at 2, 7, and 14 days. Similar color intensity could be seen in the ASA and nHAP group at all time points. Interestingly, the ASA-nHAP group exhibited the most augmented color intensity. It could be concluded that the hydrogel scaffolds promoted MC3T3-E1 cell’s osteogenic differentiation in the early stage, especially the ASA-nHAP group.

#### 3.5.2 Calcium nodule expression analysis

To further investigate the mineralization effect, the expression of calcium nodules was also detected. Figures 5B,C shows ARS staining and semi-quantitative results after 7, 14, and 21 days. What was obvious was that the more and larger red calcium nodules in the experimental groups, *versus* the control group. Clusters of calcium nodules even appeared in the ASA-nHAP group at 21 days. Also, no significant difference could be observed between the ASA group and the nHAP group. Consistent with the staining results, the analysis of the semi-quantitative results displayed significantly higher OD values of the experimental groups than the control group. It could be found that the most notable OD value is in the ASA-nHAP group. The mineralization of the extracellular matrix demonstrated that hydrogel scaffolds promoted late osteogenic markers’ expression.

#### 3.5.3 Osteogenesis-related gene expression analysis

To further verify the superiority of hydrogel scaffolds on osteogenic differentiation of MC3T3-E1 cells, the expression of osteogenesis-related genes such as RUNX2, OCN, and OPN were assessed, whose high expression was the primary condition for maturation and mineralization of osteoblasts. As shown in Figures 5D–F, the relative expression of RUNX2, OCN, and OPN of the experimental groups were higher than the control group (*p <* 0.05, *p <* 0.01) at 14 days. The ASA-nHAP group exhibited the highest gene expression. In detail, there was about a 2.5-times higher expression level of RUNX2 in the ASA-nHAP group when compared with the control group. As for OCN and OPN, it was remarkably increased in the ASA-nHAP group by offering a 5.3-times and 2.7-times than the control group, respectively. These results supported that the fabricated hydrogel scaffolds could reinforce the expression of the osteogenesis-related genes, especially for the ASA-nHAP group.

### 3.6 Anti-inflammatory property analysis

**FIGURE 6:**
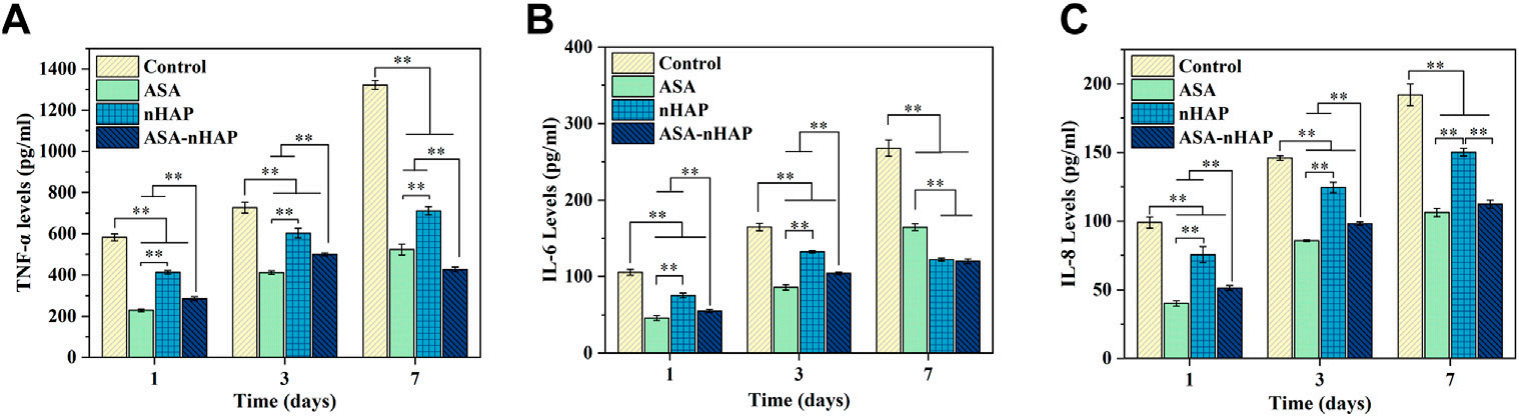
Expression of the inflammatory mediators of MC3T3-E1 cells in the inflammatory environment after 1, 3, and 7 d **(A)** TNF-α. **(B)** IL-6. **(C)** IL-8. (**p < 0.01).

ELISA was used to detect the anti-inflammatory level of the hydrogel scaffolds. As shown in Figures 6A–C, compared with the control group, the expression of TNF-α, IL-6, and IL-8 in experimental groups were lower (*p* < 0.01) at every time point. The expression of inflammatory cytokines in the control group increased over time. The increase in quantity in experiment groups was much smaller than in the control group (*p* < 0.01). Further analysis revealed that among the three experiment groups, the nHAP group had a higher level of inflammatory cytokines expression (*p* < 0.01). In the ASA group, the expression of inflammatory cytokines was significantly lower. These results indicated that the addition of aspirin and nano-hydroxyapatite gave the hydrogel scaffold an excellent anti-inflammatory effect.

## 4 Discussion

It is a superior protocol and also a great challenge that fill bone defects and promote osteoblasts’ function with bone tissue engineering materials in a special microenvironment. The design and preparation of bionic hydrogel scaffolds not only should optimize the physical, chemical, and mechanical characterization, but also improve the biological functions. To meet the need of multifunctional bionic materials, aspirin and nano-hydroxyapatite were introduced into PVA/Gel/SA hydrogel solution. Based on previous work, a dual cross-linking approach with cyclic freeze-thaw and CaCl_2_ immersion was adopted (Xu et al., 2019). The results of drug release properties, cell growth, osteogenic differentiation, and anti-inflammatory properties suggested that both aspirin and nano-hydroxyapatite are essential for the biological function of hydrogel scaffolds. Meanwhile, the synergistic biological functions to promote cell osteogenic differentiation and reduce the expression of inflammatory mediators of aspirin and nanohydroxyapatite were verified.

The interaction between molecules from different components can form new chemical structures or cause the movement of chemical bonds during the cross-linking process. FTIR analysis showed that in the hydrogel scaffold, the characteristic peaks appeared at 3,277 cm^−1^, 1734 cm^−1^, 1,627 cm^−1^, 1,550 cm^−1^, 1,024 cm^−1^, and 1,019 cm^−1^ were due to electrostatic interactions and the formation of new hydrogen bonds among raw materials during cyclic freeze-thaw (Yang et al., 2020; Zhang et al., 2021). The addition of nano-hydroxyapatite created the potential to improve the cross-link density, and the physical crosslinking made full preparation for the ideal structure of the hydrogel scaffold. The chemical cross-linking method formed a firm “egg-shell” structure, which made the molecules bound more tightly, and further enriched the 3D structure. The biological activity of the scaffolds is related to the crystal structure of the organic components. Based on XRD analysis, the results verified that the ordered arrangement of the original molecular chains and oxygen functional groups of PVA and aspirin changed, so their crystalline state disappeared in hydrogel scaffolds (Zhang et al., 2019; Maneewattanapinyo et al., 2020). The crystalline structure of nano-hydroxyapatite was not affected, which in favour to improve the mechanical strength of the hydrogel scaffold. Meanwhile, the more obvious crystalline strength of the ASA-nHAP group verified the drug-loaded ability of nano-hydroxyapatite. Some studies have reported SA, Gel, and PVA were preferred materials for the preparation of drug-loaded hydrogel scaffolds, but the bioactivity of PVA/Gel/SA hydrogel scaffold mixed with aspirin and nano-hydroxyapatite is unclear. In this study, the promoting vitality and proliferation results may be linked to the thermosensitive Gel, which is morphologically reversible during the extract process at 37°C and rich in amino acids (Zhang et al., 2021). The excellent biocompatibility of SA and PVA is also certified (Xu et al., 2019). Among the three experiment groups, the lower OD value of the nHAP group could be explained by the weakened cell viability after they devoured the nano-hydroxyapatite particles. The aspirin-loaded hydrogel could promote cell proliferation (Zhang et al., 2022). The more remarkable proliferative impact of the ASA group implied that the positive effect of aspirin compensated for the negative effect caused by nano-hydroxyapatite (Ren et al., 2018; Zhang et al., 2018). Taken together, these findings indicated that hydrogel scaffolds could effectively activate MC3T3-E1 cells and promote cell growth, which rationalized the feasibility of further studies.

During bone regeneration, not only the nutrient transport and metabolite removal, but also bio-mechanical support are influenced by the microstructure and porosity of the hydrogel scaffold (Patel et al., 2019). The graded multi-network microstructure is more suitable for cells, tissues, blood vessel ingrown and new bone formation (Zhao et al., 2022). Extensive experimental evidence demonstrated that the suitable pore size for cell adhesion and survival is 100–500 μm. The ideal porosity is 50%–90%. Moreover, smaller pore size and penetrated channel facilitate signal transduction between cells (Murphy et al., 2010; Perez and Mestres, 2016). In this study, the ideal multi-network pore structure, morphology, size, and varied orientation of the hydrogel scaffold were acquired and superior to the results of previous studies (Xu et al., 2020; Sreekumaran et al., 2021). Meanwhile, the cell-scaffold and cell-cell interaction are affected by the microstructure, surface roughness, and structural stability of the hydrogel (Schaap-Oziemlak et al., 2014; Xia et al., 2020). The compressive strength of cancellous bone was reported in the range of 0.2–4 MPa, and the compression modulus is between 20–400 Mpa (Bobbert et al., 2017). The tested compression strength of the ASA-nHAP group was 2.39 ± 0.16 MPa, and the compression modulus was 21.28 ± 3.62 MPa. They were matched to the bone tissue according to the previous literature (Goto et al., 2021). The ideal mechanical strength could be attributed to the following two reasons. Firstly, the nano-hydroxyapatite with high specific surface energy not only increased the thickness of the porous wall, but also reduced the brittleness of the nano-hydroxyapatite (Rezwan et al., 2006; Pina et al., 2015). Secondly, the Ca^2+^ released from nano-hydroxyapatite increased the cross-linking degree of SA and the formation of hydrogen-bond (Liu et al., 2020a; Suvarnapathaki et al., 2020). More importantly, it is reported that the hydrogel scaffold with excellent mechanical performance can satisfy the support demand against external stress and speed up bone formation with the stress change in the microenvironment (Chaudhuri et al., 2016). In this study, the microstructure and mechanical strength of the prepared hydrogel scaffolds provided the possibility for the growth of MC3T3-E1. The observations of the cell adhesion assay proved that the fabricated hydrogel scaffold created a favourable environment for cell proliferation. Compared with the ASA group and the nHAP group, the ASA-nHAP group attracted more cells and increased the connection between cells and cells, or the cells and their surroundings. Compared with the smooth pore wall, the rough crystal structures of nano-hydroxyapatite and the loaded aspirin could provide a focal point for the pseudopods of the MC3T3-E1 cells, which was more favourable for cell growth and crawl along the surface of the hydrogel scaffold (Hao et al., 2021). These results indicated that the hydrogel scaffold coated with nano-hydroxyapatite could provide a rougher surface and a bone-friendly environment for cell growth.

The degree of cross-linking between raw materials and the compactness of the structure are key considerations (Ozeki and Tabata, 2005), which can affect the swelling and degradation behavior of the hydrogel scaffold. During the swelling and degradation process, the hydrogel scaffold can promote cell adherence and proliferation, and perform an osteogenic function in the inflammatory environment (Akhlaq et al., 2021; Shaabani et al., 2021). Eventually, the scaffold is gradually replaced by neogenetic bones. The water-induced swelling and degradation process is linked to the breakage of polymeric chains within the biopolymer. Hence, it is critical to take the type, proportion, and cross-linking method of raw materials into consideration (Ji et al., 2020). The swelling performance of gelatin hydrogel can reach up to 1,000%, however, its stability is too poor to meet the needs of bone tissue regeneration (Zhang et al., 2019). Despite the outstanding hydrophilic properties of SA, Gel, and PVA, the suitable swelling rate of 397.71% of the ASA group manifested that the design and fabrication of the hydrogel scaffold in this study were rational. As for the nHAP and ASA-nHAP group, the dense microstructure also reduced the space where water molecules could pass, therefore, acquiring a slightly decreased swelling rate of 349.18% and 352.30%, which were consistent with the reported research (Shuai et al., 2021). Excessive swelling rate may even damage the soft tissue, increasing the risk of exposure and failure of bone healing. The internal spatial structure of hydrogel scaffolds will also change during the swelling process. Too small swelling rate makes the hydrogel scaffolds unable to absorb enough blood and provide more adequate space. Moreover, the appropriate swelling property would make the hydrogel scaffold readily adjust to the shape and volume of the bone defect area, filling the defect as soon as possible. According to the result of *in vitro* degradation rate of the nHAP and ASA-nHAP group, they could meet the requirements of bone regeneration and the osseointegration of the implant (Esposito et al., 2006). This also verified that the homogeneously dispersed nano-hydroxyapatite was tightly connected to the sites from organic components with hydrogen bonds and increased the stability of the microstructure (Uemura et al., 2019). Although the degradation rate in all groups was faster in the first 7 days, it may facilitate the release of aspirin and maintain the effective drug concentration (Tao et al., 2020). These findings fully validated that a dual-crosslinked hydrogel scaffold incorporated with aspirin and nano-hydroxyapatite made it possible to fabricate a more stable and robust material.

To regulate the inflammatory microenvironment and ensure the osteoblasts’ viability, it is advisable to use a hydrogel scaffold with a porous structure and drug binding sites as sustained release systems, and exert multi-function at different bone regeneration stages (Mauri et al., 2016). Compared to the ASA group, the current results of the continued release time of the ASA-nHAP group was 312 h. The cumulative drug release rate was 82.75%. The results were similar to the porous aspirin-loaded nanocomposite films reported in a previous study, and indicated that the hydrogel scaffold was able to retain the drug molecules without excessive loss of aspirin during the preparation process (Ji et al., 2016). As the FTIR results showed, aspirin was immobilized *via* the physical method in a 3D network structure, and nano-hydroxyapatite increased this wrapping efficiency. This not only ensures a stable chemical structure, but also significantly improves the drug release kinetics. As a result, we obtain smaller drug burst release rates and smoother release profiles. Notably, the special particle size, morphology, and crystalline state of nano-hydroxyapatite particles gave the hydrogel scaffold the potential to absorb and bind drug molecules without changing pharmacokinetic structure (Raina et al., 2020; Liu et al., 2022). When analyzed from the microstructure level, the dense and complicated polymeric chains formed by the nano-hydroxyapatite also could improve drug release behavior. In conclusion, the present results indicated that the incorporation of nano-hydroxyapatite can significantly improve the characterization of the hydrogel scaffold. Although the effect of aspirin on the structure of hydrogel scaffold was not significant, it was of great value in improving bioactivity.

Since bone repair is a complex process, it involves not only the reconstructive microenvironment, but also the inflammatory microenvironment caused by the body’s immunity and surgical microtrauma. Inflammation can stimulate the body’s immune system and promote the clearance of foreign body, but may destroy microenvironmental homeostasis. However, in the early period after biomaterial implantation, excessive inflammatory mediators produced by the foreign body rejection can disrupt osteoblast function, leading to unsatisfactory bone regeneration effect or even failure. Aspirin, bioactive nanoparticles, and Ca^2+^ released from the hydrogel scaffold can serve as major elements to promote MC3T3-E1 cells’ osteogenic differentiation. Aspirin could exert an osteogenic effect by activating the Wnt/β-catenin pathway, and increasing telomerase activity (Yamaza et al., 2008). On the surface of contained-aspirin titanium, MC3T3-E1 cells had a more pronounced osteogenic differentiation effect (Ren et al., 2018). Moreover, animal study has also demonstrated that aspirin-loaded titanium implants could treat aseptic loosening and promote osseointegration (Wei et al., 2020). Similar to the previous studies, the ASA and ASA-nHAP group showed excellent ALP activity and expression of calcium nodules. Bone tissue regeneration materials containing nano-hydroxyapatite have had comprehensive applications according to previous studies (Liao et al., 2021). The Ca^2+^ and PO_4_^3-^ released from nano-hydroxyapatite are the favorite elements for osteoblasts, which can regulate the proliferation, migration, and differentiation of osteoblasts (Kargozar et al., 2019). Therefore, the excellent results of the nHAP group and ASA-nHAP group may be explained by the hypothesis that Ca^2+^ and PO_4_^3-^ provided a suitable osteogenic differentiation microenvironment for MC3T3-E1 cells (González Ocampo et al., 2019). The qRT-PCR assay further confirmed the osteogenic differentiation effect of hydrogel scaffolds and the synergistic function of aspirin and nano-hydroxyapatite. RUNX2 reportedly is particularly important in early osteogenesis, as it is the central control gene of the osteoblast phenotype (Maji et al., 2020). OCN and OPN are the representative genes in the later osteogenic differentiation (Maji et al., 2020). The increased expression levels of related genes in the experiment groups, especially the ASA-nHAP group, demonstrated that scaffold loaded with nano-hydroxyapatite and aspirin presented excellent osteogenic differentiation capacity. Inflammatory mediators such as TNF-α, IL-6, and IL-8 can cause fever and participate in the body’s inflammatory response and immune response, which are involved in the bone destruction process (Fong et al., 2008; Gazivoda et al., 2009). The lower expression of inflammatory cytokines of experiment groups was related to the release of aspirin, Ca^2+^ and nano-hydroxyapatite. Previous literature has demonstrated that aspirin can reduce the expression of TNF-α and IFN-γ, and block the adverse effect of the NF-κB signaling pathway on osteogenesis (Clària and Serhan, 1995). The formation and resorption of bone are associated with the presence of inflammatory LPS. The Ca^2+^ can decrease the stability and amphiphilic of LPS. The ELISA results of the nHAP group and ASA-nHAP can be explained by structural modifications of LPS due to the interaction of nano-hydroxyapatite and its molecular components, inhibiting the formation of links with reactive groups of LPS and its inflammatory activity (Martínez-Sanmiguel et al., 2019). This study supports evidence of previous observations that aspirin can prevent apoptosis and inhibit inflammation (Kang et al., 2021; Yu et al., 2021), and create a friendly microenvironment (Liu et al., 2020b). Studies have confirmed that small amounts of inflammatory mediators not only maintain osteoblast viability, but also stimulate the body’s osteogenic mechanisms and accelerate bone formation (Tang et al., 2014). Thus, it is imperative to promote bone repair by regulating the expression of inflammatory cytokines and the function of the osteoblast through hydrogel scaffolds. What must be emphasized is that the ASA-nHAP hydrogel scaffold prepared in this study has excellent bionic characterization, biocompatibility, osteogenic potential, and anti-inflammatory property.

Although the multifunctional biomimetic hydrogel scaffold loaded with aspirin and nano-hydroxyapatite prepared in this study can provide a bone-friendly microenvironment and improve osteoblast activity, there are still some shortcomings. The synergistic osteogenic mechanism of aspirin and nano-hydroxyapatite is lacking, which will be benefit to further *in vivo* studies and clinical translation. However, we also made some meaningful contributions. We suggest that the design of future bioactive biomaterials should focus on a bone-friendly microenvironment which involves the regulation of the microenvironment and the improvement of the osteoblast activity and differentiation functionton to accelerate bone regeneration. It is important to consider not only the bionic structure, but also that can modulate biological processes, which involve overcoming early inflammatory responses and safely transitioning to regeneration.

## 5 Conclusion

In this work, a novel multifunctional-bionic hydrogel scaffold was designed and fabricated with a dual-crosslinking approach combined with aspirin and nano-hydroxyapatite to provide a promising osteogenic and anti-inflammatory treatment strategy for bone tissue regeneration. The characterization of hydrogel scaffolds, and their effects on cytotoxicity, osteogenic differentiation, and anti-inflammatory property were evaluated. The cyclic freeze-thawing approach created firm hydrogen bonds and electrostatic interaction among raw materials, following a secondary chemical cross-linking to form a stronger “egg-shell” structure. This method sustained the physical and biological characteristics of the raw materials, creating a bionic structure. The ASA-nHAP group preserved or even optimized the mircostructure, porosity, mechanical strength, swelling property, degradation property, and drug-release property to some extent. The ASA-nHAP group performed best in promoting MC3T3-E1 cell proliferation, adhesion, osteogenic differentiation, and anti-inflammation *in vitro* investigation. In conclusion, the simultaneous incorporation of aspirin and nano-hydroxyapatite into a hydrogel scaffold is a promising strategy for the preparation of multifunctional bone tissue engineering materials.

## Data Availability

The original contributions presented in the study are included in the article/Supplementary Material, further inquiries can be directed to the corresponding author.
