# Corrigendum: Osteogenic and anti-inflammatory effect of the multifunctional bionic hydrogel scaffold loaded with aspirin and nano-hydroxyapatite

**DOI:** 10.3389/fbioe.2023.1179873

**Published:** 2023-03-30

**Authors:** Shaoping Li, Yundeng Xiaowen, Yuqing Yang, Libo Liu, Yifan Sun, Ying Liu, Lulu Yin, Zhiyu Chen

**Affiliations:** ^1^ Key Laboratory of Stomatology in Hebei Province, Hospital of Stomatology Hebei Medical University, Shijiazhuang, China; ^2^ College of Dentistry, Hebei Medical University, Shijiazhuang, China

**Keywords:** aspirin, multifunctional hydrogel scaffold, sustained release, tissue engineering, nano-hydroxyapatite

In the published article, there was an error in Figure 2 as published. The image selection and order were incorrect. The corrected Figure 2 and its caption appear below.

**FIGURE 2:**
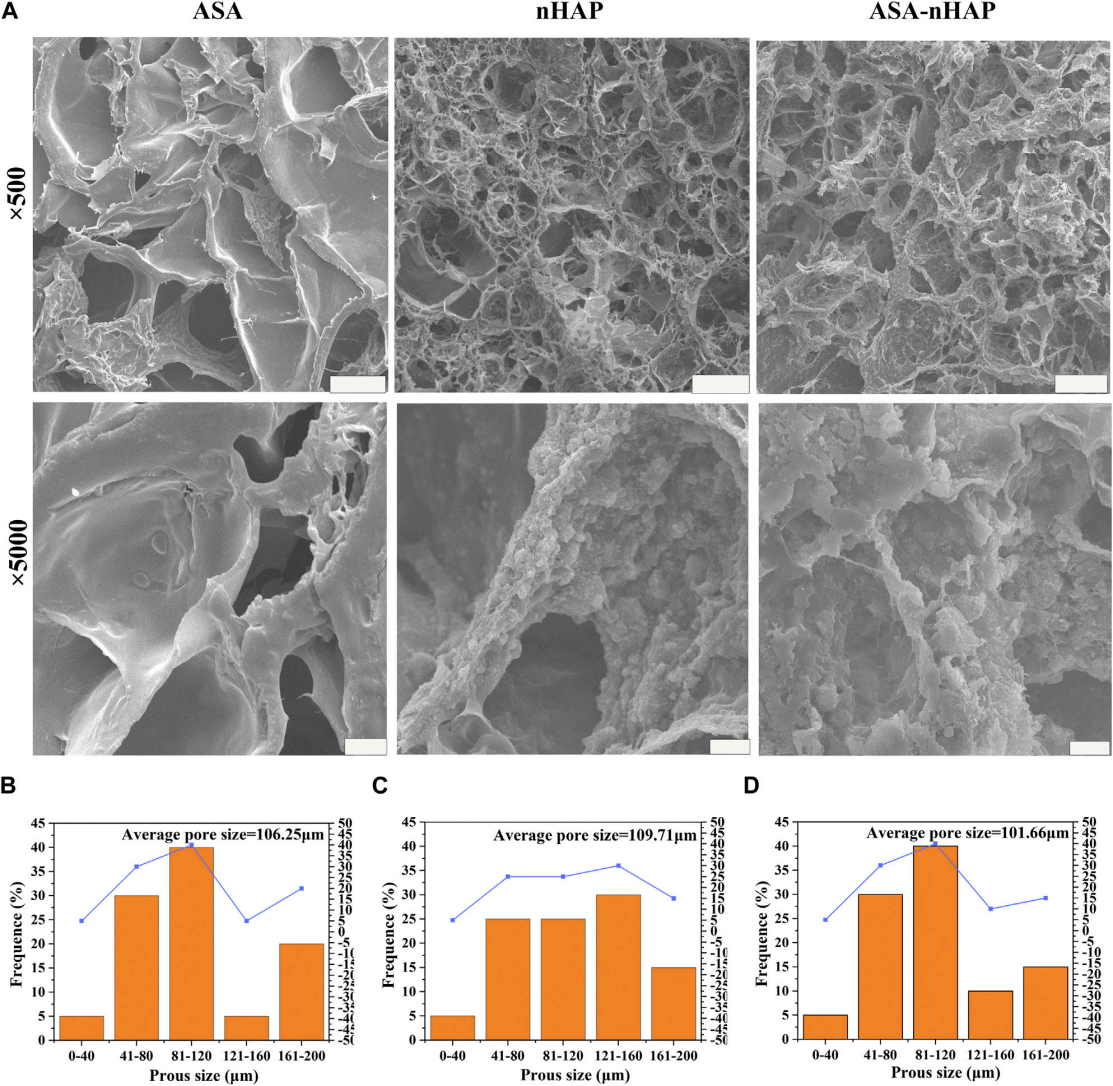
Microstructure of hydrogel scaffolds. **(A)** SEM images of the ASA group, nHAP group, and ASA-nHAP group. The scale bar for low-magnification images is 100 μm; the scale bar for high-magnification images is 10 μm. **(B–D)** Pore size distribution pattern of the ASA group, nHAP group, and ASA-nHAP group.

The authors apologize for this error and state that this does not change the scientific conclusions of the article in any way. The original article has been updated.

