# Supplementary figures and images for: Osteogenic and anti-inflammatory effect of the multifunctional bionic hydrogel scaffold loaded with aspirin and nano-hydroxyapatite
Source: Front Bioeng Biotechnol. 2023 Jan 24;11:1105248. doi: 10.3389/fbioe.2023.1105248 (PMC9902883; doi:10.3389/fbioe.2023.1105248)

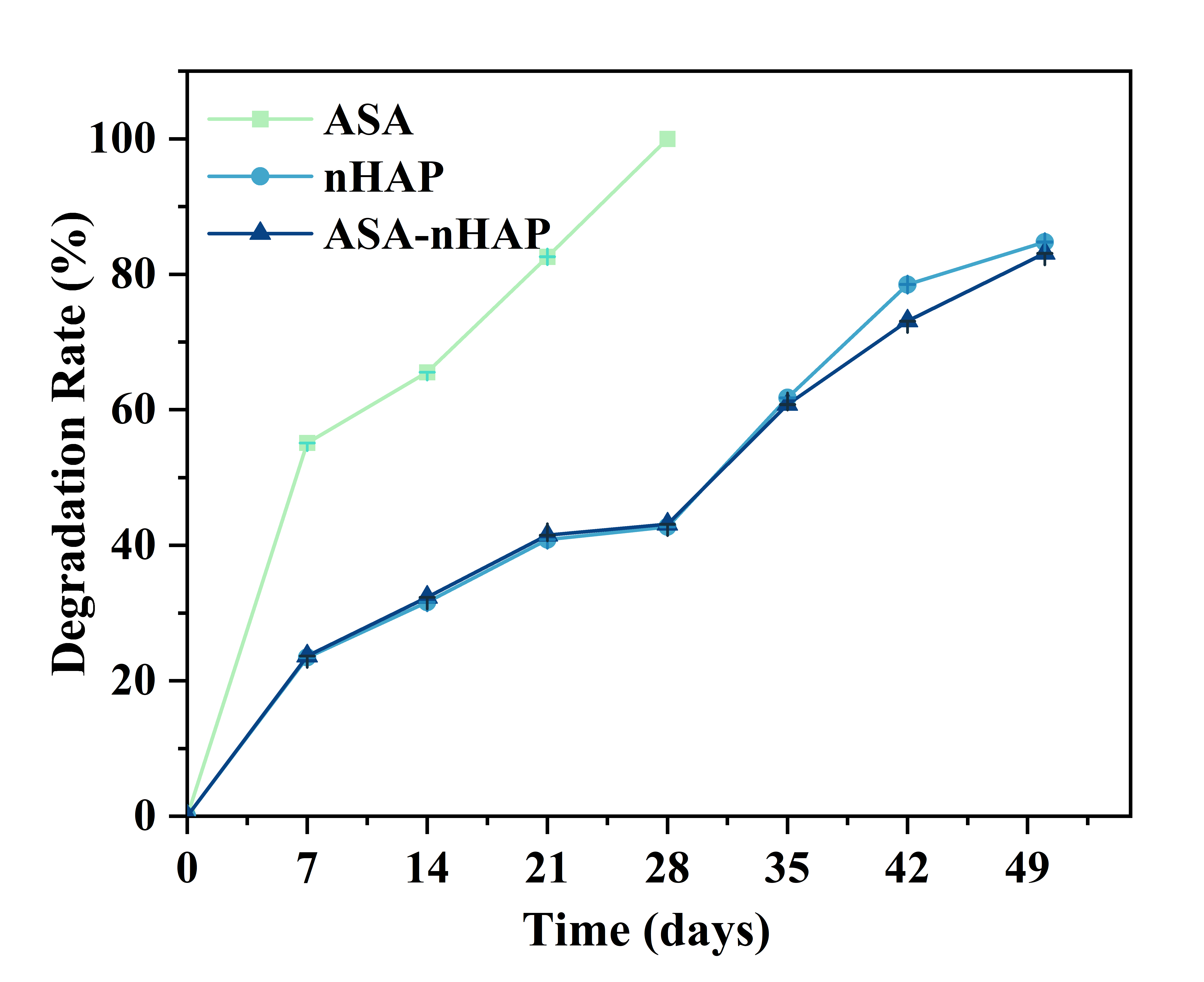

Supplement: Supplementary file 1 [file Image2.TIF]

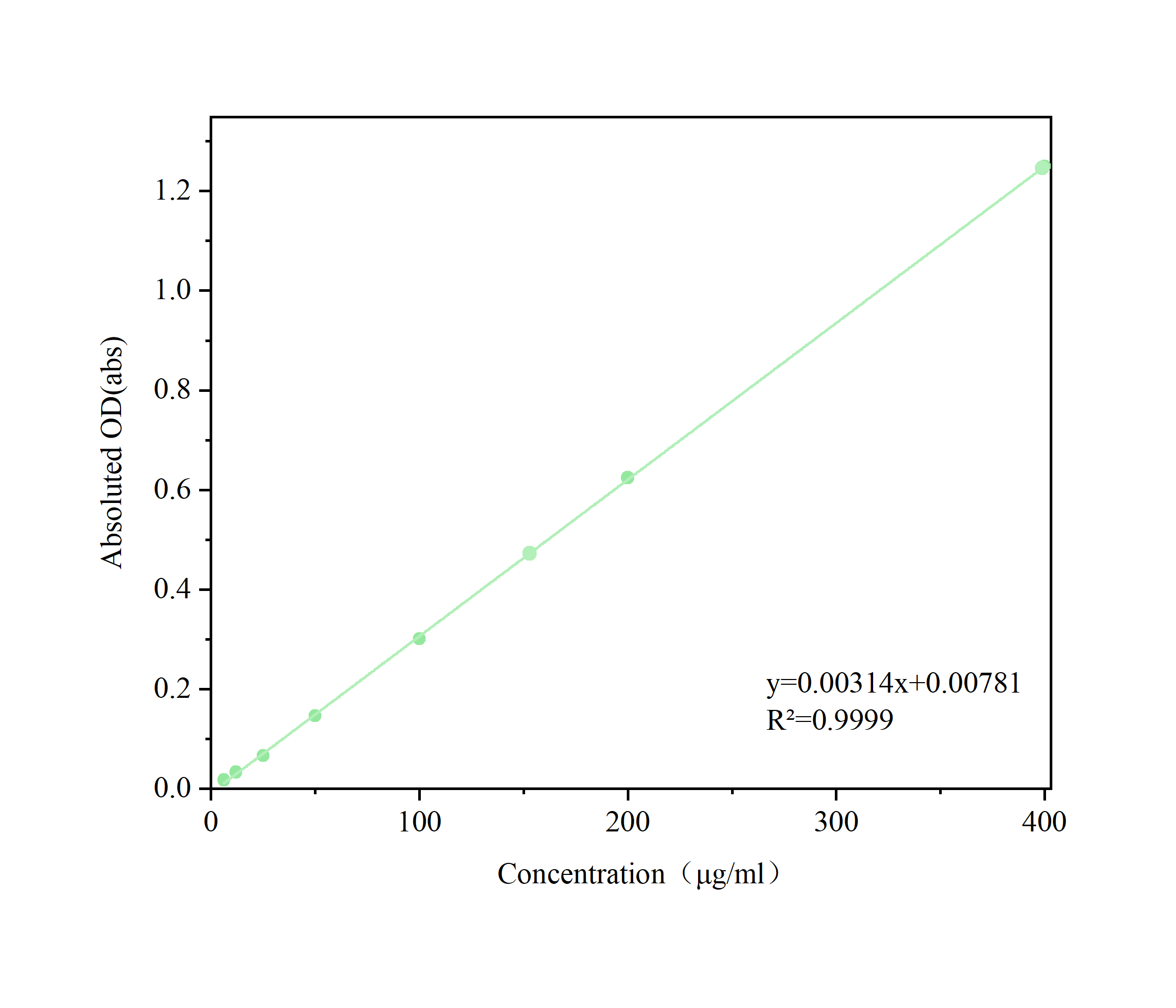

Supplement: Supplementary file 2 [file Image1.TIF]
